# Effect on Reaction Time in Primary Hypothyroid Patients Before and After Thyroxin Treatment

**DOI:** 10.7759/cureus.26074

**Published:** 2022-06-19

**Authors:** Jyoti S Kale, Nilesh T Katole, Sonali B Rode, Shubhada A Gade

**Affiliations:** 1 Department of Physiology, Datta Meghe Medical College, Datta Meghe Institute of Medical Sciences (DMIMS) Deemed to be University (DU), Nagpur, IND; 2 Department of Pharmacology, Datta Meghe Medical College, Datta Meghe Institute of Medical Sciences (DMIMS) Deemed to be University (DU), Nagpur, IND; 3 Department of Pharmacology, Jawaharlal Nehru Medical College, Datta Meghe Institute of Medical Science, Nagpur, IND

**Keywords:** central nervous system, thyroxin, visual reaction time, auditory reaction time, hypothyroid

## Abstract

Introduction

Thyroid hormones play a crucial role in the proper functioning of the nervous system. Both T3 and T4 hormones have many significant actions on the neuromuscular system and brain. In hypothyroid patients, various neurological signs and symptoms such as weakness, fatigue, paresthesias, arthralgias, etc. may be seen. Reaction time is a good indicator of the processing of the central nervous system. So, our study aims to observe the change in reaction time in hypothyroid patients as compared to the control group. And to understand, if some difference is observed, how does it change after treatment with thyroxin in a hypothyroid group.

Materials and methods

This study was conducted at the tertiary care teaching hospital in the Vidarbha region. In this study, 40 newly diagnosed primary hypothyroid patients (including males and females), in the age group of 20 to 45 years, and whose thyroid-stimulating hormone (TSH) levels were between 10 and 50 mIU/L and free T4 levels below the normal level were included. A suitable comparable control group of the same demographic parameter was selected. Reaction time was taken before the start of thyroxin treatment in both groups, and results were analyzed by using an unpaired t-test. The reaction time of the hypothyroid group was again measured after eight weeks of the start of thyroxin treatment, it was compared with the initial reaction time, and data were compared by using the paired t-test.

Result

In the hypothyroid group as compared to the control group both auditory and visual reaction times were significantly on the higher side (p<0.05). Also, there was a significant improvement in reaction time after the start of thyroxin treatment (p<0.05), which suggests improvement in CNS activity in hypothyroid patients after initiation of therapy.

Conclusion

Thyroid hormones play a crucial role in the proper functioning and processing of the central nervous system. Due to this reason, reaction time in hypothyroid patients was on the longer side, showing a slowing of the nervous system when reacting to a specific stimulus. After thyroxin treatment for a sufficient period, reaction times were of shorter duration as compared to before the start of thyroxin, which shows well-recovered nervous activity. Therefore, reaction time is not only used as a handy tool to identify early central nervous system manifestations of hypothyroidism but also used to monitor response to treatment.

## Introduction

Reaction time can be defined as the time required for applying a stimulus and the initiation of a response to that stimulus [1]. It shows how fast the conduction of neurophysiological and cognitive information processes is on the application of a stimulus to one’s sensory system. Thus, the reaction time is the combination of receiving information (may be visual or auditory), the processing, decision-making, and response to that stimulus, or the execution of a motor act. All these processes follow one another and form a reaction time (RT) [2-4].

Thyroid hormones (triiodothyronine (T3) and thyroxin (T4)) play a significant part in the tissue development and metabolism of the body. These hormones exert many effects on the neuromuscular system and brain. That’s why hypothyroidism may lead to various neurological signs and symptoms. Muscular weakness, fatigue, muscular and mental sluggishness, weight gain, etc. are some of the common signs of hypothyroidism [5].

Reaction time is an important parameter, used for many years to study the processing of the information in the central nervous system, which is necessary to prepare and execute a response to a received stimulus [6-7]. As hypothyroidism affects the nervous system, it causes neurological signs and symptoms. Reaction time can be an efficient tool to assess neurological signs in hypothyroid patients. Therefore, our study aims to observe the effect on reaction time in hypothyroid patients as compared to control and if there is any delay, whether the reaction time improves after treatment with thyroxin.

## Materials and methods

This is a case-control study. The study was conducted in a tertiary care hospital in the Vidarbha region of India. The duration of the study was six months. This study included 40 newly diagnosed primary hypothyroid patients (both males and females). Forty subjects of similar demographic parameters were chosen as the control group: those without any vision and hearing disease, not taking any medication, and not practicing any exercise or yoga therapy. Smokers, alcoholics, and those having chronic illnesses like diabetes and hypertension were excluded from the study. Patients in the age group between 20 and 45 years were included. The TSH level was between 10 and 50 mIU/L and the free T4 level was below normal (normal value of free T4 in an adult: 0.9-1.7 ng/dL). Institutional ethics committee approval (institutional ethics committee, Datta Meghe Medical College, approval number IEC/2021/145) was obtained and written informed consent was taken from each subject before the commencement of study. Those having other acute or chronic diseases were excluded from the study. Pregnant and lactating women were excluded. Reaction time was taken before the start of thyroxin treatment and again after eight weeks of thyroxin treatment. Both groups’ socioeconomic variables were comparable.

Reaction time was measured in a quiet room of the physiology department between 10 am and 12 pm. A reaction time machine records auditory time for low and high pitch sounds. For visual reaction time, it records red, green, and yellow colors as low and high-intensity stimuli for each respective color. Subjects were sensitized to machines and environment by giving satisfactory practice of recording so they could perform their best. During the actual recording, the subject was asked to pay full attention to receiving stimulus. They were asked to press the button with the index finger of the dominant hand, as they will receive a stimulus. Five recordings were taken. The mean of the middle three recordings was taken as the final reading. Time was recorded in milliseconds.

Statistical analysis

A student's unpaired t-test was used to compare reaction time between the control and hypothyroid groups and a paired t-test was used to compare reaction time before and after thyroxin treatment in the hypothyroid group.

## Results

As shown in Table 1, as compared to the control group, both auditory and visual reaction times in the hypothyroid group were significantly on the higher side (p<0.05). In the control group, it was found that auditory reaction time was on the lower side as compared to visual reaction time. For visual reaction time, for red light color, the reaction time is more delayed as compared to for yellow and green light.

**Table 1:** Reaction time in the hypothyroid patient group and control group RT – reaction time, LS – low-intensity stimulus, HS – high-intensity stimulus *p < 0.05 Mean ± SEM

Stimulus for RT	RT (in milliseconds) in the hypothyroid group	RT (in milliseconds) in the control group
Auditory RT – LS	365 ± 45	198 ± 34*
Auditory RT – HS	312 ± 32	192 ± 26*
Visual RT Red – LS	412± 25	252 ± 31*
Visual RT Red – HS	390 ± 35	240 ± 28 *
Visual RT Green – LS	450 ± 22	295 ± 25 *
Visual RT Green – HS	422 ± 40	275 ± 22 *
Visual RT Yellow – LS	450 ± 41	273 ± 28 *
Visual RT Yellow – HS	442 ± 35	264 ± 25 *

As shown in Table 2, in hypothyroid patients, after thyroxin treatment, auditory reaction time, both for low and high-intensity stimuli, was significantly reduced. Similar findings were seen for visual reaction time as well. For all three colors, namely, red, green, and yellow, and for both low and high-intensity stimuli, a significant difference (p<0.05) was seen.

**Table 2:** Reaction time in hypothyroid patients before and after thyroxin treatment RT – reaction Time, LS – low-intensity stimulus, HS – high-intensity stimulus *p < 0.05 Mean ± SEM

Stimulus For RT	RT (in milliseconds) before Thyroxin treatment	RT (in milliseconds) after Thyroxin treatment
Auditory RT – LS	365 ± 45	290 ± 29*
Auditory RT – HS	312 ± 32	242 ± 23*
Visual RT Red – LS	412± 25	335 ± 31 *
Visual RT Red – HS	390 ± 35	281 ± 32*
Visual RT Green – LS	450 ± 22	382 ± 24 *
Visual RT Green – HS	422 ± 40	362 ± 22 *
Visual RT Yellow – LS	450 ± 41	329 ± 27 *
Visual RT Yellow – HS	442 ± 35	324 ± 25 *

## Discussion

Thyroid hormones play an important role in proper cognitive development as well as the proper functioning of the nervous system. They are involved directly in interaction with intrinsic regulatory circuits or indirectly with systemic effects. For example, effects on the circulatory system or metabolic pathways. Due to these close relations with the nervous system function, disturbances of thyroid function with low levels of thyroid hormones are associated with a wide spectrum of neurological signs and symptoms, including mood and cognitive disorders, headache, tremors, muscle weakness, movement disorders, sluggish reflexes, and inattentiveness, etc. [8]. 

Reaction time is an important indicator of the processing ability of the central nervous system. It is used in physiology to assess sensory-motor performance. Reaction time is the time between the onset of the stimulus and the movement of response. Reaction time is also used in psychology, mental chronometry, and in athletes to assess performance [9]. Therefore, we used a reaction time tool to determine the status of central nervous system processing in hypothyroid individuals.

Hypothyroidism is accompanied by the mental and physical slowing of an individual [10]. Table 1 shows that there is a prolongation in both auditory and visual reaction times in the hypothyroid group than in the control subjects. Prolonged reaction time shows reduced nervous system performance [11]. The reasons for this can be explained by the regulation of many functions and processes of the nervous system by thyroid hormone [5,12]. Reduced levels of thyroid hormones significantly affect the functioning of the nervous system. In hypothyroidism, there is a generalized decrease in basal metabolic rate, sensory perception is decreased, and neuronal transmission is delayed. This is due to alteration in carbohydrate, lipid, and protein metabolism [13]. Irmgard D and Sivaraj et al. showed in their study that after four weeks of thyroid hormone withdrawal, there is a decrease in visual perception speed, speed of speech, and visual-spatial orientation, which shows a decline in central neuronal information processing [14].

We found auditory reaction times are longer compared to visual reaction times. Ears are faster to respond to a given stimulus and process its message faster along the auditory pathways toward the brain as compared to receptors and optic pathways of the eyes [15]. This probably accounts for the difference in the auditory and visual reaction times.

Visual reaction time to red light is on the lower side as compared to green and yellow lights. This can be explained as, when shifting from one color stimulus to another, it changes in both luminance and chromaticity. The visual reaction time of a performer in detecting this chromatic change depends on the luminance change and is regulated by Pieron’s law [16]. Also, Cattell had found that the reaction time for red and green colors was almost similar but for yellow colors was on the longer side [17-18].

Both (auditory and visual) reaction times were significantly (p<0.05) improved after treatment with thyroxin as shown in Table 2. We treated all hypothyroid patients with thyroxin for an intermediate duration (eight weeks). Reaction time was again measured after eight weeks. It was significantly improved as compared to the initial stage. Similar findings were reported by Shah SH and Nahar PS [13] that audiovisual reaction time was on the higher side in primary hypothyroidism, as well as in hyperthyroidism patients, and significant improvement was seen after correction of hormonal imbalance. Similar findings were also reported by Vedavathi KJ [19] and Shubham G et al. [20].

In the initial phase of hypothyroidism, it is mostly asymptomatic or may have only mild symptoms like easy fatigability, weakness, cold intolerance, constipation, weight gain, dry skin, etc. while more serious symptoms are seen at a later stage like voice changes, abnormal menstrual disturbance, depression, etc. CNS manifestations are also manifested at a very late stage, hence even patients will not notice those changes in the early stage. However, prolongation of reaction time is seen in the early phase, before the appearance of major symptoms, hence reaction time can be an important clinical tool for a physician, not only for the diagnosis of hypothyroidism but also for monitoring treatment output [21].

Limitation of the study

The results of this study are difficult to extrapolate to the community, considering the smaller sample size of the study.

## Conclusions

Thyroid hormones play a crucial role in the proper functioning and processing of the central nervous system. Due to this reason, reaction time in hypothyroid patients was on the longer side, showing a slowing of the nervous system to react to a specific stimulus. After thyroxin treatment for a sufficient time, the reaction time became of shorter duration as compared to before the start of thyroxin, which showed well-recovered nervous activity. Reaction time can be a useful clinical tool to diagnose nervous system manifestations during the initial phase of hypothyroidism. Also, it may be useful to monitor response to thyroxin therapy.
